# Plasma retinol, beta-carotene and vitamin E levels in relation to the future risk of breast cancer.

**DOI:** 10.1038/bjc.1988.49

**Published:** 1988-02

**Authors:** N. J. Wald, A. Nicolaides-Bouman, G. A. Hudson


© The Macmillan Press Ltd., 1988

LETTER TO THE EDITOR

### Plasma retinol, beta-carotene and vitamin E levels in relation to the future risk of breast cancer

**Sir** – Russell and his colleagues present results in this issue (Russell *et al.*, 1988) that apparently differ from those published in 1984 by Wald *et al.* (1984). The latter found that women who subsequently developed breast cancer (cases) had significantly lower vitamin E levels than matched controls, but Russell and his colleagues found no significant difference. The studies related to different women drawn from the same population (but with sera collected about 10 years apart).

Since cases were compared to controls in each study, systematic changes in sample or assay technique cannot explain the discrepancy between the two studies. Early cancer appears to affect serum vitamin E levels (Wald *et al.*, 1987), but in the study by Wald and his colleagues (1984), the interval between serum collection and the diagnosis of breast cancer was sufficiently long (mean 4.5 years) to make this explanation unlikely.

Although when we reported our serum vitamin E and breast cancer results in 1984, we believed that the quality of the stored serum samples from cases and controls was sufficiently sound to permit comparison of the concentrations of micronutrients in the samples, storage was only at −20°C and no records were kept of the number of times the samples had been withdrawn from storage. We cannot therefore exclude the possibility that vitamin E degradation may have occurred, and done so to a greater extent in the samples from the cases than in those from the controls. Our recently published negative results on serum vitamin E and cancer in men (Wald *et al.*, 1987) and the present results on breast cancer reported by Russell and his colleagues prompted us to re-examine this possibility. We therefore reassayed the retinol, beta-carotene and vitamin E levels in those of the original serum samples that were still available and compared the current values with those in 1981 (and reported in 1984) to obtain an indication of the extent to which sample storage and handling between 1981 and 1986 affected the serum levels, and, by inference, how such conditions may have affected them before. We also estimated degradation in the complement component C3, which is sensitive to sample handling and storage, by immunofixation electrophoresis and densitometry.

The results of the vitamin assays are shown in Table I. There were marked declines in levels of both vitamin E and of beta-carotene between 1981 and 1986. There was also a statistically significant difference in C3 degradation between samples from cases and those from controls in 1986 ($P<0.05$); the mean percentages of degradation were 84.4% in the 30 cases and 78.6% in the 52 controls. It is possible that these results reflect differences that were present in 1981.

**Table I** Re-assay in 1986 of the 52 available serum samples from control subjects that were assayed in 1981 and published by Wald *et al.* (1984)

| | *1981* | | *1986* | |
|---|---|---|---|---|
| | *Mean* | *s.d.* | *Mean* | *s.d.* |
| Retinol ($\mu g\,l^{-1}$) | 466 | 122 | 448 | 132 |
| $\beta$-carotene ($\mu g\,l^{-1}$) | 59 | 89 | 3 | 6[a] |
| Vitamin E ($mg\,l^{-1}$) | 6.45 | 3.24 | 3.10 | 1.69[a] |

[a]Difference between 1981 and 1986, $P<0.001$, sign test.

We conclude that the results reported by Wald *et al.* (1984) may have been artefactual. This episode stresses the importance of ensuring that biological samples used for prospective studies are stored satisfactorily and that records, especially of freezing and thawing, are kept to ensure the comparability of cases and controls.

Yours etc.

N.J. Wald & A. Nicolaides-Bouman,
Department of Environmental and Preventive Medicine,
Medical College of St Bartholomew's Hospital,
Charterhouse Square,
London EC1M 6BQ,
UK.

G.A. Hudson,
Rheumatic Disease Laboratory,
Foundation for Blood Research,
Box 190,
Scarborough,
Maine,
USA.

### References

RUSSELL, M.J., THOMAS, B.S. & BULBROOK, R.D. (1988). A prospective study of the relationship between serum vitamins A and E and risk of breast cancer. *Br. J. Cancer*, **57**, 000.

WALD, N.J., BOREHAM, J., HAYWARD, J.L. & BULBROOK, R.D. (1984). Plasma retinol, beta-carotene and vitamin E levels in relation to the future risk of breast cancer. *Br. J. Cancer*, **49**, 321.

WALD, N.J., THOMPSON, S.G., DENSEM, J.W. BOREHAM, J. & BAILEY, A. (1987). Serum vitamin E and subsequent risk of cancer. *Br. J. Cancer*, **56**, 69.


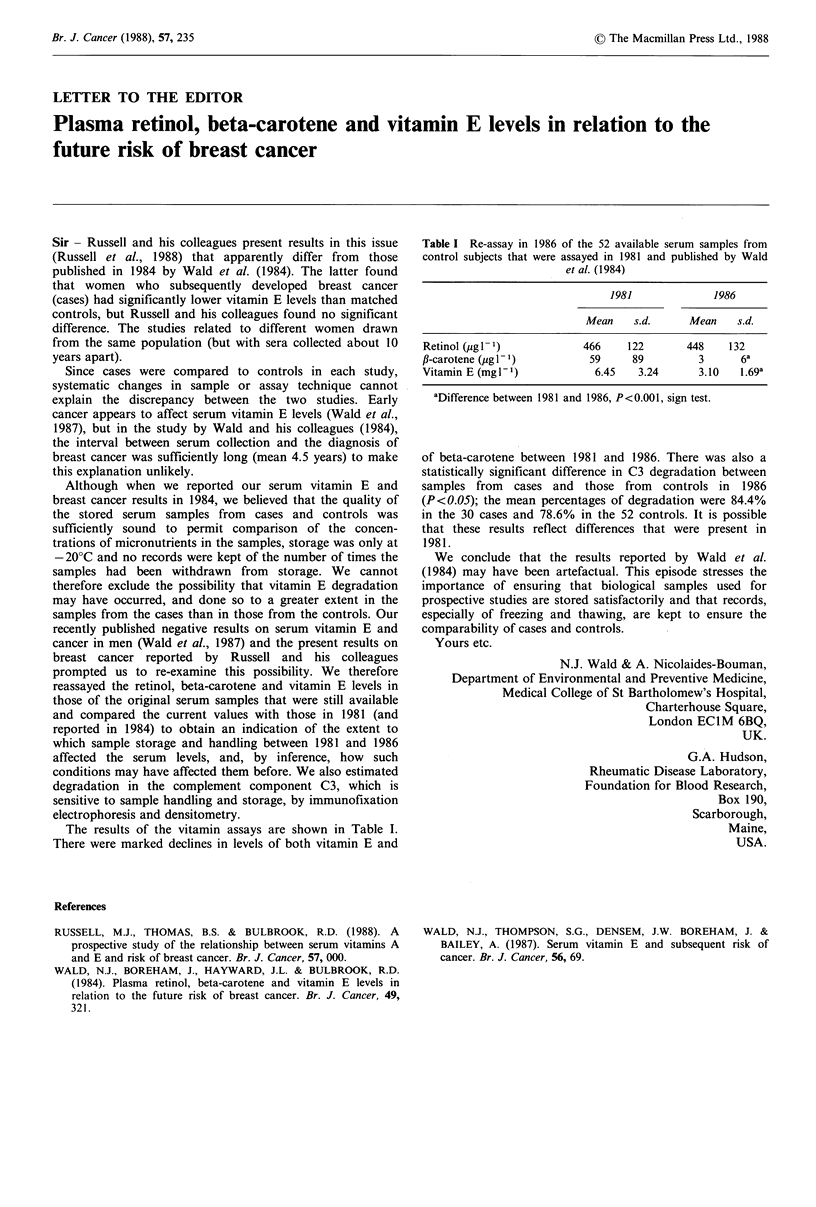


## References

[OCR_00115] Wald N. J., Boreham J., Hayward J. L., Bulbrook R. D. (1984). Plasma retinol, beta-carotene and vitamin E levels in relation to the future risk of breast cancer.. Br J Cancer.

[OCR_00123] Wald N. J., Thompson S. G., Densem J. W., Boreham J., Bailey A. (1987). Serum vitamin E and subsequent risk of cancer.. Br J Cancer.

